# Dietary Mushroom Intake May Reduce the Risk of Breast Cancer: Evidence from a Meta-Analysis of Observational Studies

**DOI:** 10.1371/journal.pone.0093437

**Published:** 2014-04-01

**Authors:** Jiaoyuan Li, Li Zou, Wei Chen, Beibei Zhu, Na Shen, Juntao Ke, Jiao Lou, Ranran Song, Rong Zhong, Xiaoping Miao

**Affiliations:** State Key Laboratory of Environment Health (Incubation), MOE (Ministry of Education) Key Laboratory of Environment & Health, Ministry of Environmental Protection Key Laboratory of Environment and Health (Wuhan), and Department of Epidemiology and Biostatistics, School of Public Health, Tongji Medical College, Huazhong University of Science and Technology, Wuhan, China; National Cancer Center, Japan

## Abstract

Epidemiological studies have investigated the potential anticancer effects of mushroom intake. This review aims to clarify the evidence on the association of dietary mushroom intake with breast cancer risk and to quantify its dose-response relationship. Relevant studies were identified by a search of PubMed, Web of Science and Google Scholar up to December 31, 2013. Observational studies with relative risks (RRs) or hazard ratios (HRs) or odd ratios (ORs) and 95% confidence intervals (CIs) of breast cancer for three or more categories of mushroom intake were eligible. The quality of included studies was assessed by using Newcastle-Ottawa Scale. A dose-response meta-analysis was performed by utilizing generalized least squares trend estimation. Eight case-control studies and two cohort studies with a total of 6890 cases were ultimately included. For dose-response analysis, there was no evidence of non-linear association between mushroom consumption and breast cancer risk (*P* = 0.337) and a 1 g/d increment in mushroom intake conferred an RR of 0.97 (95% CI: 0.96–0.98) for breast cancer risk, with moderate heterogeneity (*I^2^* = 56.3%, *P* = 0.015). Besides, available menopause data extracted from included studies were used to evaluate the influence of menopausal statues. The summary RRs of mushroom consumption on breast cancer were 0.96 (95% CI: 0.91–1.00) for premenopausal women and 0.94 (95% CI: 0.91–0.97) for postmenopausal women, respectively. Our findings demonstrated that mushroom intake may be inversely associated with risk of breast cancer, which need to be confirmed with large-scale prospective studies further.

## Introduction

Breast cancer is the most common cancer and the leading cause of cancer death for female in both developed and developing countries, accounting for 23% of the total new cancer cases and 14% of the total cancer deaths in 2008 [1]. The high prevalence and incidence have led to a large public health burden all over the world, thus more attention should be paid to the primary prevention of breast cancer.

Lifestyle factors are considered to play an important role in the prevention of breast cancer since they could be modified [2]. Intriguingly, many lifestyle factors make different effects on breast cancer risk according to different menopausal status [3], [4]. Menopausal status was closely related to breast cancer, with the mediation of hormone levels change in women. The risk factors of premenopausal breast cancer were also not completely as same as that of postmenopausal breast cancer [3], suggesting underling etiologies may be different. In addition, the prognosis and treatment options of breast cancer depend on menopausal status. Exemplified by the fact that aromatase inhibitors had been particularly given to the hormone therapy of postmenopausal hormone-dependent breast cancer [5]. So, it's important to take menopausal status into account, if possible, in breast cancer research.

As essential components of lifestyle, diet-related factors are thought to account for about 30% of cancers in developed countries [6]. Various daily foods, such as cruciferous vegetables [7], fish [8], coffee [9], tea [10], and soy products [11], have been indicated to be correlated with the risk of breast cancer by numerous studies. Mushroom, as a common vegetable supplied in daily diet worldwide, contains an abundance of pharmaceutically active compounds. The most investigated compound derived from mushroom is polysaccharide, which has antitumor and immunomodulating properties [12]. Laboratory studies have demonstrated the antitumor activity of specific mushrooms, in particular, medicinal mushrooms both *in vivo* and *in vitro* [13], [14]. Moreover, adjuvant treatments with medicinal mushroom extracts were shown to be capable of improving prognosis of breast cancer [15], [16], though their exact effectiveness need to be confirmed.

Several studies reported an adverse association of edible mushroom intake with the risk of breast cancer [17]–[22]. However, some other researches failed to observe the significant protective effect of mushroom consumption against breast cancer [23]–[25]. Given the inconsistent results of the existing literature and limited sample sizes of individual studies, we conducted a meta-analysis of observational studies with the following objectives: (1) to summarize the evidence on the association between edible mushroom consumption and risk of breast cancer and quantify the potential dose-response pattern; (2) to examine whether the relationship is affected by menopausal status.

## Methods

### Search strategy

We performed a systematic literature search on PubMed, Web of Science, and Google Scholar up to December 31, 2013 using the following key words: “mushroom” or “fungi” and “breast cancer”, “breast carcinoma”, “breast tumor” or “breast tumour”. The reference lists of selected articles were also scrutinized to obtain additional pertinent publications. Only articles written in English were included.

### Study selection

Studies were eligible if they met the following criteria: (1) the study had a case-control or cohort design; (2) the exposure of interest was dietary intake of edible mushroom; (3) the outcome was the occurrence of breast cancer; (4) the study provided relative risks (RRs), hazard ratios (HRs) or odds ratios (ORs) with 95% CIs for ≧ 3 categories of exposure; (5) the number of cases and the total subjects or follow-up person-years for each category of mushroom intake were reported or derivable by published data. If an article reported results for premenopausal and postmenopausal women respectively, we separated this article into two independent studies by menopausal status.

### Data extraction

The following information were extracted from each included study: the first author's name, year of publication, study population, study design, age of participants, number of cases, menopausal status, daily mushroom consumption, OR, RR or HR with corresponding 95% CI for each category of mushroom consumption and adjusted potential confounders. The effect size that reflected the greatest degree of adjustment for potential confounders was included.

### Quality assessment

The Newcastle-Ottawa Scale (NOS) [26] was used to assess the quality of the eligible studies. Each study included in the meta-analysis was judged on three broad dimensions: the selection of the study subjects (four items), the comparability of the study populations (one item) and the ascertainment of the exposure in case-control studies or outcome of interest in cohort studies (three items). A study can be awarded a maximum of one star for each numbered item within the selection and exposure or outcome categories, but two stars for item of comparability. Thus, the total score for a single study ranges from zero to nine. A study was considered to be of high quality if scored seven or more stars.

### Statistical analysis

The RR and HR are assumed to approximate the OR because of the low incidence of breast cancer [27], thus we combined the RR and HR with OR in current meta-analysis and reported the summary effect size as RR for simplicity.

For each study, the median or midpoint of upper and lower boundaries was assigned as the average intake of mushroom in each category. If the upper boundary of the highest category was not provided, we assumed that the upper boundary had the same amplitude as the closest category. We performed a dose-response model by using general least-squares trend estimation as described by Greenland and Longnecker [28]. This approach which based on constructing approximate covariance estimates for the log relative risks and estimating corrected linear or non-linear trend using general least squares has been widely applied in previously published meta-analyses [29]–[32]. We also established a restricted cubic spline model to explore the potential non-linear relationship [33]. Cubic splines are generally defined as piecewise-polynomial line segments whose function values and first and second derivatives agree at the boundaries where they join. The boundaries of these segments are called knots, and the fitted curve is continuous and smooth at the knot boundaries [34]. In this meta-analysis, we established a cubic spline model with 3 knots at 25%, 50% and 75% percentiles of the distribution and a *P* value for non-linearity was calculated by testing the null hypothesis that the coefficient of the second spline was equal to zero.

The between-study heterogeneity was assessed by the Cochran *Q* test and *I^2^* statistic and it was considered significant if *P*<0.10 for *Q* statistic or *I^2^*>50%. When there was significant heterogeneity detected, data from included studies were combined by random-effects model; otherwise, the fixed-effects model was utilized. Meta-regression was initially conducted to find the source of heterogeneity, and then subgroup analysis was carried out if feasible. Sensitivity analyses were executed by deleting each study in turn to estimate the influence of individual studies on the pooled estimate. Besides, we evaluated publication bias by Begg's and Egger's regression tests.

All statistical analyses were conducted with Stata 10.0 and a *P*<0.05 was considered statistically significant unless noted otherwise.

## Results

### Literature search and study characteristics

**Figure 1:**
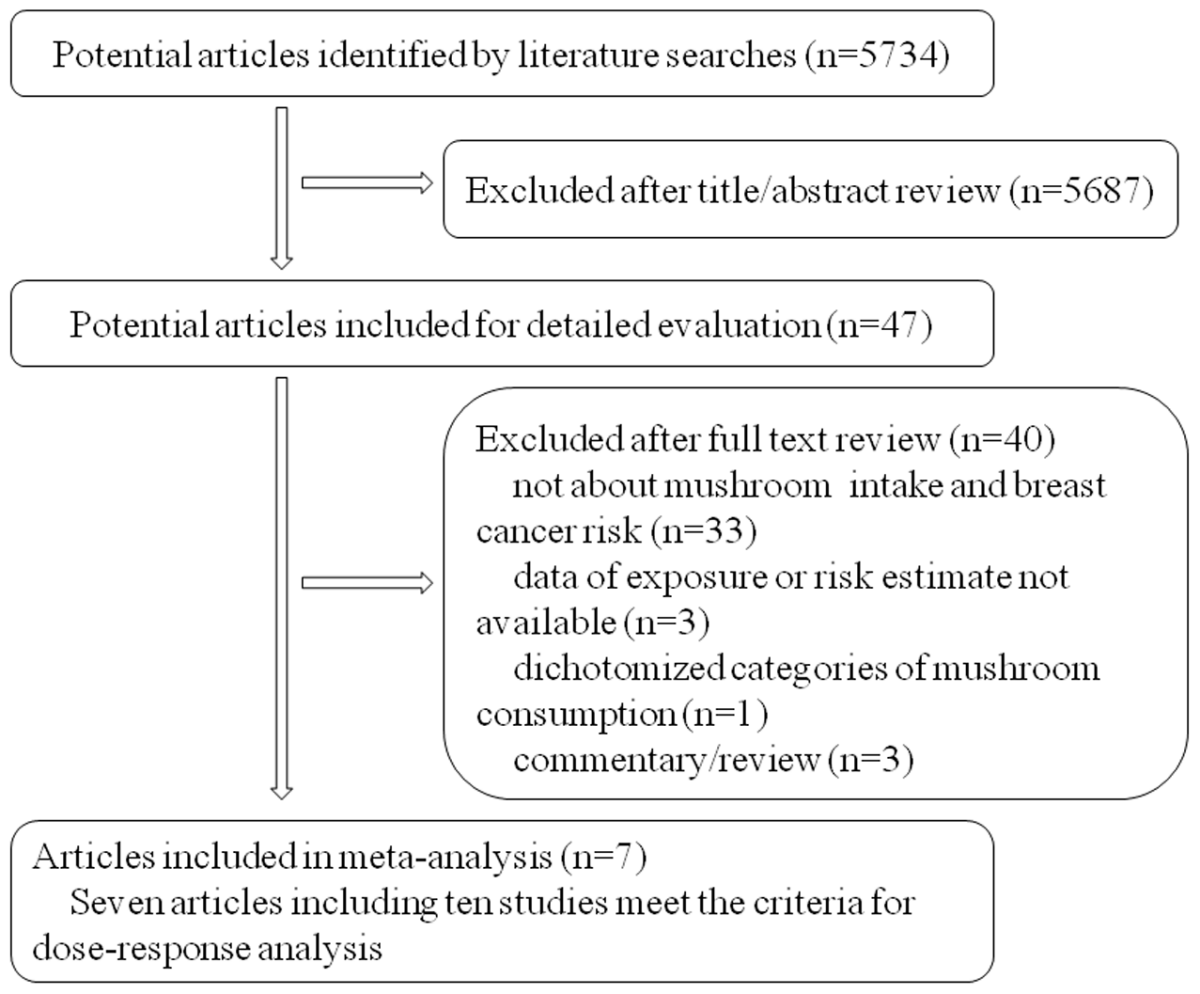
Flow chart of articles' selection.

The flow chart of literature search was shown in Figure 1. Initially, 5734 articles were identified by literature search, of which 5687 articles were excluded after review of titles or abstracts. Forty articles were further excluded due to the following reasons: no report of the association between mushroom intake and breast cancer risk (n = 33); data of exposure or risk estimates not available (n = 3); dichotomized categories of mushroom consumption (n = 1); comment or review (n = 3). Finally, seven original articles [17]–[20], [22], [24], [25] that met our inclusion criteria were included in this study. Three articles provided independent data by menopausal status, thus were considered apart. Therefore, ten independent studies were eventually applied for the dose-response meta-analysis. Of the included studies, eight adopting case-control design with 2313 cases and 2387 controls were conducted in Asian and two adopting cohort design with 4,577 cases and 1,748,623 follow-up person-years were conducted in Europe. All of the ten studies scored six or more stars, and seven out of the ten studies were of high quality (NOS score ≧ 7). The main characteristics of included studies were summarized in Table 1.

**Table 1:** Characteristics of studies included in the meta-analyses.

First author	Year	Study population	Study design	Age	No. of cases	Menopausal status	NOS score	Mushroom consumption	Risk estimates	Adjusted confounders
										
Hong [17]	2008	Korea	CC	30–65 y	235	Premenopause	7	0 g/d	reference	Education, family history of breast cancer, regular exercise, BMI, current smoker, current drinker, current multivitamin supplement, number of children, energy, carbohydrate, soy protein, vitamin E and folate
								2.8 g/d	0.38 (0.18–0.80)	
								4.9 g/d	0.51 (0.24–1.10)	
								15.1 g/d	0.60 (0.30–1.23)	
								30 g/d	0.44 (0.19–1.00)	
Hong [17]	2008	Korea	CC	30–65 y	127	Postmenopause	7	0 g/d	reference	Education, family history of breast cancer, regular exercise, BMI, current smoker, current drinker, current multivitamin supplement, number of children, energy, carbohydrate, soy protein, vitamin E and folate
								1.9 g/d	0.88 (0.24–3.21)	
								3.2 g/d	0.20 (0.05–0.71)	
								8.0 g/d	0.44 (0.14–1.38)	
								15.1 g/d	0.16 (0.04–0.54)	
Zhang [18]	2009	China	CC	20–87 y	479	Premenopause	7	0 g/d	reference	Age, residential area, education, BMI, age at menarche, OC, HRT, family history, total energy intake, alcohol consumption, active smoking, passive smoking, tea drinking and physical activity
								0∼2 g/d	0.86 (0.59–1.23)	
								2∼10 g/d	0.71 (0.48–1.01)	
								>10 g/d	0.34 (0.22–0.52)	
Zhang [18]	2009	China	CC	20–87 y	212	Postmenopause	7	0 g/d	reference	Age, residential area, education, BMI, age at menarche, OC, HRT, family history, total energy intake, alcohol consumption, active smoking, passive smoking, tea drinking and physical activity
								0∼2 g/d	0.97 (0.57–1.65)	
								2∼10 g/d	0.99 (0.56–1.75)	
								>10 g/d	0.35 (0.17–0.70)	
Zhang [19]	2009	China	CC	25–70 y	438	Not specified	6	<0.8 g/d	reference	Age at menarche, BMI, history of benign breast disease, family history, physical activity, passive smoking and total energy intake
								0.8∼2.5 g/d	0.95 (0.66–1.38)	
								2.5∼7.1 g/d	0.69 (0.45–1.04)	
								>7.1 g/d	0.65 (0.43–0.98)	
Shin [20]	2010	Korea	CC	25–77 y	210	Premenopause	7	<2.6 g/d	reference	Age, BMI, family history, dietary supplements, education, job, smoking, alcohol intake, physical activity, age at menarche, parity, total energy intake, and vegetable intake
								2.6∼5.4 g/d	0.76 (0.36–1.60)	
								5.4∼11.4 g/d	0.76 (0.34–1.70)	
								>11.4 g/d	0.35 (0.13–0.91)	
Shin [20]	2010	Korea	CC	25–77 y	148	Postmenopause	7	<2.6 g/d	reference	Age, BMI, family history, dietary supplements, education, job, smoking, alcohol intake, physical activity, age at menarche, parity, energy intake, vegetable intake and postmenopausal hormone use
								2.6∼5.4 g/d	1.20 (0.54–2.63)	
								5.5∼11.4 g/d	0.77 (0.32–1.81)	
								>11.4 g/d	0.74 (0.23–2.33)	
van Gils [25]	2005	10 European countries	Cohort	25–70 y	3505	Not specified	6	2.2 g/d	reference	Energy intake, alcohol intake, saturated fat intake, height, weight, age at menarche, parity, OC, HRT, menopausal status, smoking status, physical activity, and education
										
								2.8 g/d	0.91 (0.80–1.05)	
								3.5 g/d	0.87 (0.76–1.01)	
								4.2 g/d	1.01 (0.88–1.17)	
								5.0 g/d	0.98 (0.85–1.14)	
Masala [24]	2012	Italy	Cohort	36–64 y	1072	Not specified	7	<0.4 g/d	reference	Weight, height, education, number of children, age at menarche, menopausal status, energy intake except alcohol, alcohol intake, current use of hormone therapy, smoking status, physical activity
								0.4∼0.9 g/d	0.93 (0.77–1.12)	
								1.0∼1.9 g/d	0.82 (0.67–1.01)	
								2.0∼4.0 g/d	0.94 (0.79–1.12)	
								>4.0 g/d	0.85 (0.69–1.05)	
Mizoo [22] *	2013	Japan	CC	>20 y	464	Not specified	6	2.6 g/d	reference	Age
								15.4 g/d	0.73 (0.54–0.98)	
								33.3 g/d	0.60 (0.40–0.91)	

*: The dose of mushroom consumption in each category was calculated based on the average intake of mushroom per day in Japan.

Abbreviations: CC: case-control; NOS: Newsastle-Ottawa Scale; BMI: body mass index; OC: oral contraceptive; HRT: hormone replacement therapy.

### Dose-response analysis

**Figure 2:**
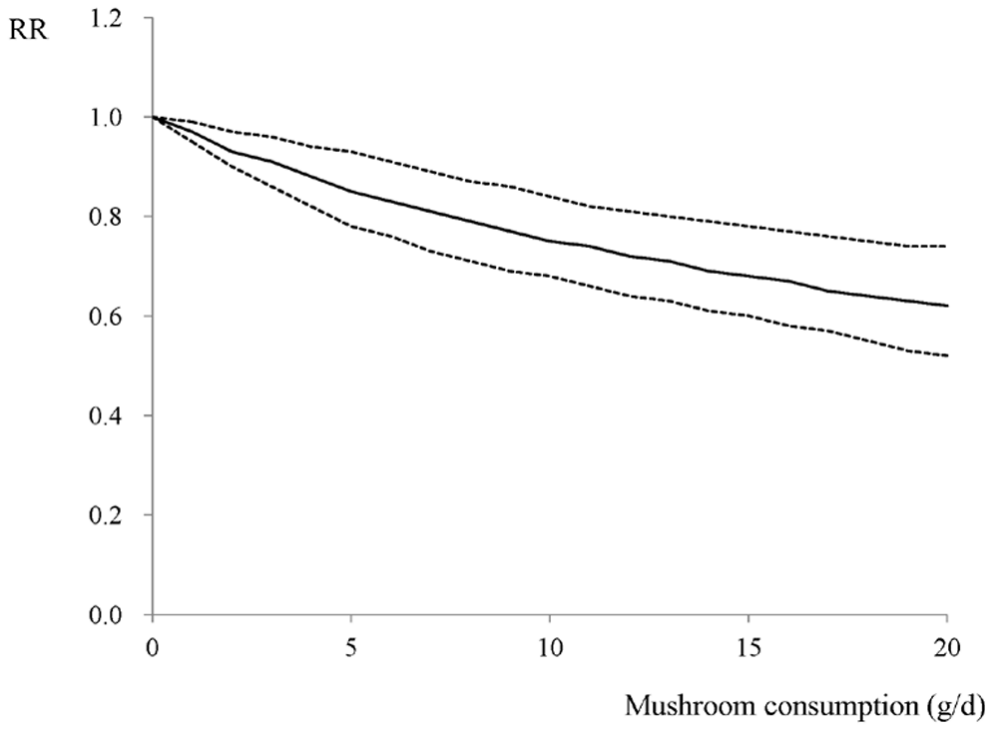
The dose-response analysis for the association of mushroom consumption and breast cancer risk, with restricted cubic splines in random-effects dose-response model. The solid line and the short dash line represent the estimated relative risks and corresponding 95% CIs, respectively.

**Figure 3:**
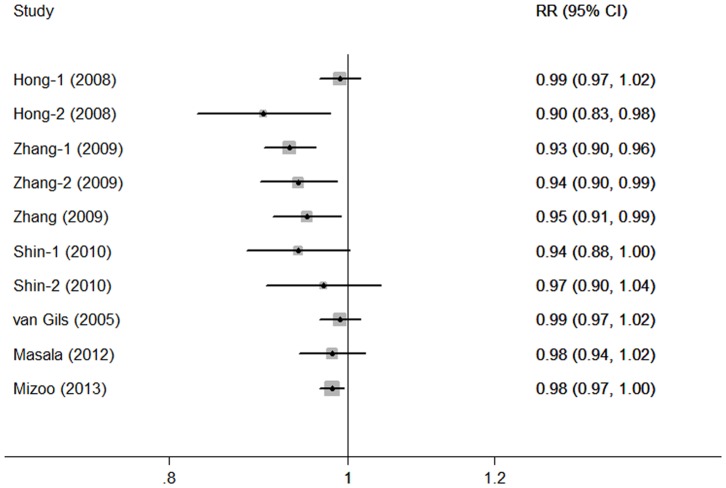
Study-specific dose-response analyses for the relationship between mushroom consumption and risk of breast cancer.

There was no evidence of significant departure from linearity among data from the 10 studies (*P* = 0.337). A 1 g/d increment in mushroom consumption conferred an RR of 0.97 (95% CI: 0.96–0.98, Figure 2) for breast cancer risk, with moderate heterogeneity (*I^2^* = 56.3%, *P* = 0.015). The study-specific RRs per 1 g/d increase in mushroom consumption were presented in Figure 3.

**Figure 4:**
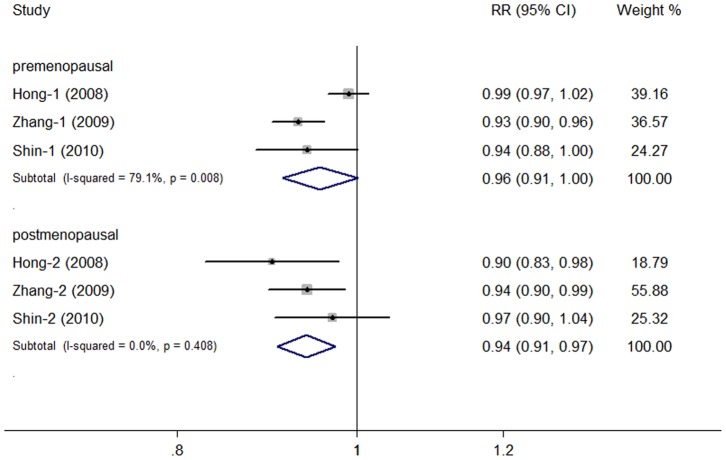
Dose-response meta-analyses for premenopausal and postmenopausal women.

Meta-analyses for premenopausal and postmenopausal women were performed separately to detect the role of menopausal status in the relationship between mushroom intake and breast cancer risk. Four studies were not included in this analysis owing to lack of data split by menopausal status. There was significant heterogeneity (*I^2^* = 79.1%, *P* = 0.008) among studies for premenopausal women, while no evidence of heterogeneity (*I^2^* = 0%, *P* = 0.408) was detected among studies for postmenopausal women. Significant associations were observed in both groups (Figure 4), with the summary RRs being 0.96 (95% CI: 0.91–1.00) for premenopausal women and 0.94 (95% CI: 0.91–0.97) for postmenopausal women.

### Meta-regression

Meta-regression was performed to explore potential sources of between-study heterogeneity. Firstly, an empty regression was run to estimate the baseline value for *tau^2^*. Then, univariate meta-regressions were successively conducted with following covariates: study population (Asian or Europe), study design (case-control or cohort), number of adjusted confounders (≧12 or <12), number of cases (≧400 or <400), whether adjusted for body mass index (BMI), whether adjusted for cigarette smoking, whether adjusted for alcohol drinking and whether adjusted for physical activity, respectively. As a result, none of these variables showed statistically significant associations in univeriate meta-regression models (*P*>0.05), suggesting that factors which mentioned above could not explain the heterogeneity among studies. Considering the negative result of meta-regression, subgroup analysis was not conducted further.

### Sensitivity analysis and publication bias

Sensitivity analysis was carried out by omitted one study at a time and calculated the combined RR for remaining studies (Table 2). The relevant between-study heterogeneity were significant (*P*<0.10) except the study conducted in premenopausal women by Zhang et al. was excluded (*I* ^2^ = 31.3%, *P* = 0.168). But after removed this study, there was still an adverse association of mushroom consumption and breast cancer risk (RR  = 0.97, 95% CI: 0.96–0.99). The results were not materially altered when other studies was deleted in turn, with RRs ranging from 0.96 (95% CI: 0.94–0.98) to 0.97 (95% CI: 0.95–0.99).

**Table 2:** Sensitivity analyses of included studies.

Study omitted	Population group	*I^2^* (%)	*P* for heterogeneity	RR* (95% CI)
Hong 2008-1	Premenopause	54.8	0.024	0.96 (0.94–0.98)
Hong 2008-2	Postmenopause	51.3	0.037	0.97 (0.95–0.98)
Zhang 2009-1	Premenopause	31.3	0.168	0.97 (0.96–0.99)
Zhang 2009-2	Postmenopause	55.1	0.023	0.97 (0.95–0.99)
Zhang 2009	Not specified	56.9	0.017	0.97 (0.95–0.98)
Shin 2010-1	Premenopause	57.2	0.016	0.97 (0.95–0.98)
Shin 2010-2	Postmenopause	59.7	0.011	0.97 (0.95–0.98)
van Gils 2005	Not specified	54.6	0.024	0.96 (0.94–0.98)
Masala 2012	Not specified	59.4	0.012	0.96 (0.95–0.98)
Mizoo 2013	Not specified	56.3	0.019	0.96 (0.94–0.98)

*: The relative risk per 1 g/d increment in mushroom consumption for breast cancer.

**Figure 5:**
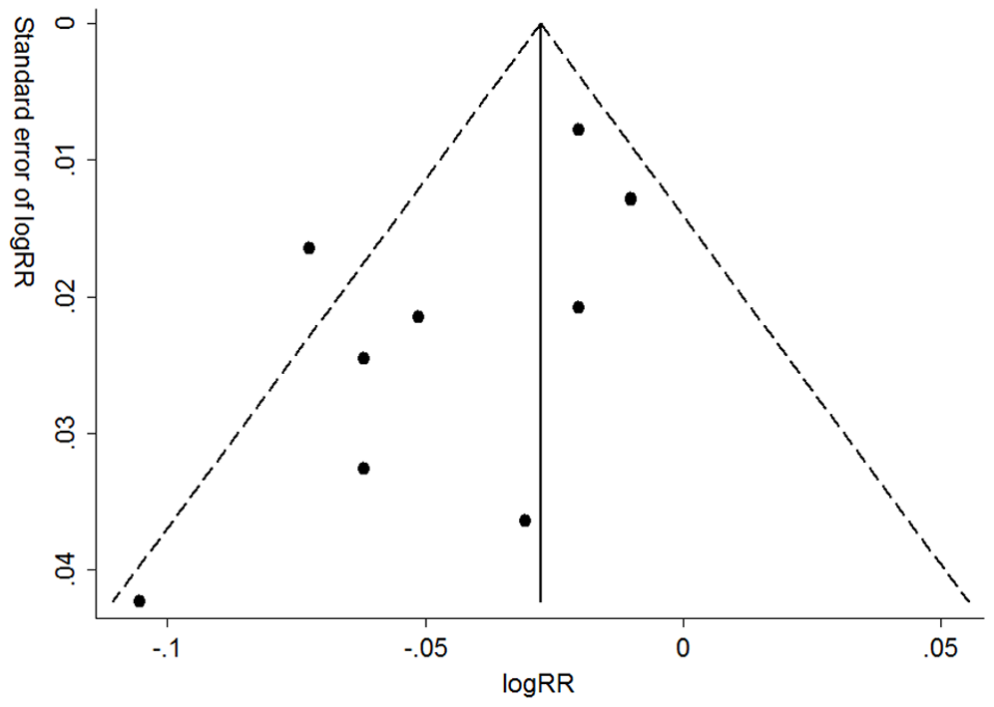
Publication bias in the studies.

For publication bias, funnel plot showed no obvious asymmetry (Figure 5). Besides, neither Egger's regression test nor Begg's test detected evidence of publication bias (*P* = 0.06 for Egger's regression test and 0.107 for Begg's test).

## Discussion

The meta-analysis including 10 eligible studies indicated a linear dose-response association between mushroom intake and risk of breast cancer, with the summary RR being 0.97 (95% CI: 0.96–0.98) for per 1 g/d increment in mushroom consumption. Besides, the protective effects of mushroom intake on risk of breast cancer were consistently exhibited in premenopausal women and in postmenopausal women.

Potential benefits to breast cancer patients taking high dosage of the extract of specific medicinal mushrooms over long term have been well-studied with positive results [35]. While benefits of oral administration of mushrooms on breast cancer risk were still unclear. In the present meta-analysis, the summary RRs of breast cancer presented a steady linear decrease with the increasing intake of edible mushroom. Extensive evidences from biological and clinical studies have addressed the most important properties of mushroom in the antitumor and immuno-modulating activities. As we know, it is actually the biologically active substances of mushroom which play a key role in various vital processes including antitumor activities [36]. The major bioactive compounds of edible mushrooms especially polysaccharides and glucan function in the antitumor activity, which were strongly supported by evidences from *in vitro* and *in vivo* experiments [37]–[40]. For example, a study reported by Jeong et al. indicated that polysaccharides isolated from *Agaricus bisporus* white button mushroom, a common edible mushroom consumed in most countries, had the ability to inhibit the growth of human breast cancer MCF-7 cells in part through activation of nuclear factor-κB with the production of p50/105 heterodimers. Additionally, in *in vivo* experiments, a reduction in tumor growth was observed when murine sarcoma 180 cells exposed to polysaccharides were implanted subcutaneously into mice [37]. Meanwhile, *Amauroderma rude*, a well-known medicinal mushroom, has been reported could inhibit cancer cell survival and induce apoptosis, and suppression of c-myc expression appeared to be associated with these effects [41]. These pre-existing researches verified the beneficial therapeutic effects of edible and medicinal mushrooms on breast cancer. However, their preventive benefits against breast cancer have not been elucidated. We supposed that polysaccharides may bring down the occurrence of breast cancer through their strikingly effects on immune system. The polysaccharides were regarded as biological response modifiers (BRMs), by which both innate and adaptive immune responses can be modulated. With different structures, polysaccharides present distinct affinities towards their specific receptors to trigger a wide spectrum of host immune responses [42], which were capable of recognizing aberrant transformed cells and eliminating tumor cells [43]. Although the present meta-analysis supported the role of edible mushrooms in the suppression of breast cancer, association studies with larger sample size and well-designed clinical studies are still warranted to further verify the significant results.

Interestingly, the health benefits of mushroom intake for breast cancer presented no difference between premenopausal and postmenopausal women in our meta-analysis. Considering only three eligible studies included in the meta-analysis, it was not very appropriate to make a conclusion about whether the linear trend between mushroom intake and risk of breast cancer influenced by the menopausal status. By now, a few epidemiological studies have investigated this association by menopausal status and almost no experimental study has been conducted for the biological function of mushroom or mushroom extracts intake on the risk of breast cancer in terms of the hormone circumstance. It was worth to note that the association between mushroom intake and risk of breast cancer stratified by menopausal status need to be further explored in large perspective studies, and the underlying mechanisms should be uncovered by functional studies.

The development of breast cancer is a multifactorial process, thus the association between mushroom and breast cancer may be confounded by many other factors [44]–[48], which possibly brought heterogeneity to the meta-analysis. Considering the between-study heterogeneity, meta-regression analysis has been conducted to search for the source of the heterogeneity. Unfortunately, no factor was identified. However, the between-study heterogeneity was disappeared after we excluded a study conducted in premenopausal women by Zhang et al. [18] in sensitivity analysis, implying the removed study might account for a proportion of heterogeneity. We speculated that the characteristics of the sub-population reported by Zhang et al. might differ from other included ones, but more detailed information was not offered in the study. Nevertheless, the sensitive analysis confirmed the stability of the significant association between mushroom consumption and breast cancer risk.

The strengths for the current meta-analysis had been summarized. To the best of our knowledge, the meta-analysis firstly systematically explored the pooled effect for edible mushroom consumption on the risk of breast cancer. Besides, our analysis precisely estimated the pooled relative risks with an application of dose-response approaches. Moreover, we have convinced that the results of our systematic meta-analysis, in essence, were stable and reliable after performing sensitivity analyses and testing the publication bias. Despite the clear strengths, some limitations should be acknowledged. First, the number of eligible studies included in the meta-analysis was relatively insufficient. Besides, despite of 70% high-quality studies, only two of the ten included studies were prospectively designed, thus additional large-scale and well-designed studies are warranted. Second, we have no opportunity to identify the source of heterogeneity because of the limited information extracted from the original studies. Oral administration of mushrooms in suppressing breast cancer involves many confounders and thus benefits of mushroom consumption are less convincing, as opposed to extracts. Although most original studies had adjusted many potential confounding factors, other heterogeneous natures of studies, such as demographic, reproductive factors and other lifestyle characteristics, possibly made effect on the current results. However, we could not perform further analysis owing to lack of detailed data. Further, we couldn't ignore the heterogeneous effects of different mushroom types since there were too many different mushroom species in different countries. It's a big challenge for us to restrict edible mushroom species. Thus our result indicated the combined effects of many edible mushrooms.

In conclusion, the results from this meta-analysis suggested that greater edible mushroom consumption may be associated with a lower risk of breast cancer. Our research provided a perspective that oral administration of mushrooms perhaps contribute to breast cancer primary prevention. Whereas available data are still sparse, the findings need to be updated and confirmed with well-designed prospective studies in future.

## Supporting Information

Checklist S1 **PRISMA checklist.** Preferred Reporting Items for Systematic Reviews and Meta-Analyses.
(DOC)
